# Molecular detection methods and serotyping performed directly on clinical samples improve diagnostic sensitivity and reveal increased incidence of invasive disease by *Streptococcus pneumoniae* in Italian children

**DOI:** 10.1099/jmm.0.2008/000935-0

**Published:** 2008-10

**Authors:** Chiara Azzari, Maria Moriondo, Giuseppe Indolfi, Cristina Massai, Laura Becciolini, Maurizio de Martino, Massimo Resti

**Affiliations:** Department of Paediatrics, University of Florence and Anna Meyer Children’s Hospital, Viale Pieraccini 24, Florence I-50139, Italy

## Abstract

The aims of this study were to evaluate the incidence of invasive pneumococcal disease (IPD) in Italian children and perform serotyping by PCR-based assays directly on clinical samples. A 1-year paediatric (0–14 years) population-based surveillance study was designed to evaluate the incidence of IPD in the province of Florence, Italy, by cultural and molecular methods. Among 92 children (80 with pneumonia, 8 with meningitis/sepsis, 4 with arthritis), 4 cases of IPD were diagnosed both by culture and real-time PCR and 18 cases exclusively by molecular methods. The sensitivity of molecular methods was significantly higher than that of cultural methods (Cohen’s *κ* 0.41; McNemar *P*=0.000008). The incidence of IPD in children below 2 years of age was 11.5/100 000 and 51.8/100 000 by cultural and molecular methods, respectively. Pneumococcal serotyping by multiplex sequential PCR was obtained in 19/22 samples. Real-time PCR and multiplex sequential PCR can be used directly on biological samples, improving the ability to diagnose IPD. The incidence of IPD appears 5–10 times higher by PCR than by cultural methods.

## INTRODUCTION

*Streptococcus pneumoniae* is a major aetiological agent for pneumonia, meningitis, otitis and sepsis, among both young children and elderly people all over the world (Black *et al.*, 2007; CDC, 1997). The organism is the cause of at least 1.2 million deaths each year in children due to sepsis, meningitis and pneumonia (CDC, 1997; Hausdorff *et al.*, 2000).

The incidence of invasive pneumococcal disease (IPD) varies across countries (Domínguez *et al.*, 2002; Smith *et al.*, 1998; Whitney *et al.*, 2003) and is probably largely underestimated since diagnosis of invasive pneumococcal infections, usually performed by culturing from whole blood or cerebrospinal fluid (CSF), requires the presence of viable pathogens in the clinical samples. Very few data are available for Italy, where the only available source is a passive surveillance system started in 1994 (National Surveillance System for Bacterial Meningitis) where both meningitis and sepsis are reported (Italian National Institute of Health, 2007; http://www.simi.iss.it/dati.htm). According to that system, the mean incidence rate for meningitis seems to be around 0.5/100 000 (Italian National Institute of Health, 2007; http://www.simi.iss.it/dati.htm). A prospective surveillance of IPD was undertaken in two Italian regions in 2001 by standard cultural methods and the highest incidence recorded for children was 6–11/100 000 for children below 2 years of age (D'Ancona *et al.*, 2005).

PCR-based assays for direct detection of pneumococci from clinical samples do not require viable bacteria and reach a very high sensitivity, representing an important tool in the diagnosis of invasive pneumococcal infections (Clarke, 2006). However, up to now, PCR-based methods on clinical samples have been used to discriminate between pneumococci and other pathogens (Corless *et al.*, 2001) but not for pneumococcal serotyping. Serotype distribution is usually monitored by serological determination of the capsular type by standard capsular tests after culture (D'Ancona *et al.*, 2005) or by molecular serotyping of isolates (Pai *et al.*, 2006; Kong *et al.*, 2006), so that pneumococcal serotyping cannot be performed on culture-negative samples.

The aim of the present study was to evaluate the incidence of IPD through a 1-year paediatric population-based surveillance study in an Italian province and to perform pneumococcal serotyping using molecular methods directly on clinical samples.

## METHODS

### Study design.

An observational, prospective, cohort study was designed to evaluate the incidence of IPD in a paediatric population in the province of Florence, Italy, in a 12-month period between 1 December 2005 and 30 November 2006. The study was performed using a molecular diagnostic method, set up in the Laboratory of Paediatric Immunology of the University of Florence, directly on biological samples.

### Patients.

All children 0–14 years referred to the Anna Meyer Children’s Hospital were considered. The Anna Meyer Children’s Hospital is the only hospital in the district of Florence in which a paediatric infectious disease unit and a paediatric intensive care unit are present, and it is the only one in the province where patients with meningitis, sepsis, arthritis or osteomyelitis as well as with complicated pneumonia are admitted. The Anna Meyer Children’s Hospital is also a third level hospital and accepts patients from all of Tuscany and other Italian regions. Thus, in order to avoid overestimation of pneumococcal incidence, all analyses were uniquely calculated on the basis of the cohort of residents in the district of Florence (Italian National Institute of Statistics, ISTAT, 2007; http://demo.istat.it/). All Florentine children with fever, leukocytosis and/or increased levels of serum C-reactive protein and a clinical diagnosis of meningitis, sepsis, complicated pneumonia, osteomyelitis or arthritis were included in the study. Sepsis was clinically suspected by the presence of previously described signs and confirmed by blood tests (Russell, 2006). Meningitis was clinically suspected by the presence of a compatible clinical syndrome and confirmed by chemical (Overturf, 2005) and microbiological CSF tests (cultural or molecular testing). Osteomyelitis or arthritis were clinically suspected by the presence of local signs (Kaplan, 2005) and confirmed by radiological procedures. Complicated pneumonia was suspected in the presence of clinical signs such as tachypnoea and pathological breath sounds (Ostapchuk *et al.*, 2004) and confirmed by X-ray consistent with pneumonia and demonstrating lobar or segmental lung consolidation with or without pleural effusion. An IPD case was defined as one in a child admitted to Anna Meyer Children’s Hospital in the study period with one of the above-mentioned diseases and a laboratory-confirmed presence of *S. pneumoniae* in whole blood and/or CSF and/or pleural fluid by cultural and/or molecular tests. Clinical conditions preceding the present episode, previous vaccinations and any underlying disease were recorded. Informed consent was obtained for their child by parents or guardians.

### Data sources.

Data concerning children 0–14 years, resident in the district of Florence, were provided by the National Institute of Statistics (Italian National Institute of Statistics, ISTAT, 2007; http://demo.istat.it/).

### Vaccination.

In the province of Florence, as well as in the whole Tuscan region, pneumococcal vaccination is not included in the vaccination schedule for the paediatric or adult population. Conjugate vaccine is sometimes suggested by individual paediatricians mainly for infants attending day-care centres; vaccination expenses (both vaccine and administration expenses) are paid by the families with a co-payment system.

### Clinical samples.

Whole blood was obtained from all children included in the study; CSF was obtained from children with a clinical suspicion of meningitis. Pleural fluid was obtained from two patients who needed a pleural drainage. Biological samples were obtained as soon as possible after hospital admission and used both for cultural testing and for molecular testing by real-time (RT)-PCR and multiplex sequential PCR.

### DNA extraction.

Bacterial genomic DNA was extracted from 200 μl of biological samples using the QIAmp DNeasy Blood & Tissue kit (Qiagen), according to the manufacturer’s instructions. 

### RT-PCR. .

Primers and probes were designed using the ABI Primer Express Software Package based on previously published *ctrA*, *bexA*, and *lytA* sequences from the meningococcal capsular transfer gene, from the *Haemophilus influenzae* capsule exporting gene and from the pneumococcal autolysin gene, respectively. The *ctrA* sequence-specific probe was FAM-labelled, the *bexA* probe was NED-labelled, and the *lytA* probe was JOE-labelled. Primer and probe sequences are shown in Table 1. The RT amplification was performed in 25 μl reaction volumes containing 2× TaqMan Universal Master Mix (Applied Biosystems); primers (for *S. pneumoniae*, *Neisseria meningitidis* and *H. influenzae* type b) were used at a concentration of 300 nM; the FAM-labelled probe was used at a concentration of 25 nM and NED- or JOE-labelled probes were used at a concentration of 50 nM. Six microlitres of DNA extract was used for each reaction. All reactions were performed in triplicate. A negative control (no template) and a positive control for each pathogen were included in every run. DNA was amplified in an ABI 7000 sequence detection system (Applied Biosystems) using the following cycling parameters: 95 °C for 10 min followed by 45 cycles of a two-stage temperature profile of 95 °C for 15 s and 60 °C for 1 min.

### Serotyping.

*S. pneumoniae* serotyping was performed on RT-PCR positive samples through a sequential multiplex PCR on DNA extracted from biological samples as for RT-PCR. Thirty-one primer pairs (Pai *et al.*, 2006; O’Halloran & Cafferkey, 2005) for serogroups/serotypes 1, 3, 4, 5, 6A/B, 7F/A, 7C/B, 8, 9V/A, 10A, 11A/D/F, 12A/B/F, 14, 15A, 15B/C, 16F, 17F, 18A/B/C/F (two pairs of primers), 19A, 19F, 20, 22A/F, 23F, 31, 33A/F, 34, 35B, 35F and 38F were grouped into nine multiplex reactions. *cpsA* (pneumococcal capsular polysaccharide synthesis gene) primers (Pai *et al.*, 2006) were included in each reaction mix as a confirmatory test; samples positive for serogroup 14 were also tested for confirmation with a different primer pair (http://www.cdc.gov/ncidod/biotech/strep/pcr.htm). The PCRs were performed in 25 μl volumes, with the mixture containing 1× PCR Master Mix (Qiagen) and primers (0.2–0.5 mM each); 5 μl DNA extract was used in each PCR. Amplification was performed in a Perkin-Elmer GeneAmp PCR system 2720 (Applied Biosystems) under the following conditions: 95 °C for 15 min followed by 35 amplification cycles of 94 °C for 30 s, 54 °C for 90 s and 72 °C for 60 s. A final hold was performed at 72 °C for 10 min. As previously described (Kaplan *et al.*, 2004), pneumococcal serotypes were classified as ‘vaccine serotype’ if they were 4, 6B, 9V, 14, 18C, 19F or 23F. They were classified as ‘potentially cross-reactive serotypes’ if they were from the same serogroups but serotype could not be identified and they were classified as ‘non-vaccine serotypes’ if samples were positive for serogroups different from those contained in the heptavalent conjugate vaccine.

### PCR product detection.

The PCR products were analysed by gel electrophoresis on 2 % NuSieve agarose gels (Cambrex Bio Science) in 1× TAE buffer. Gels were stained with ethidium bromide (0.5 μg ml^−1^), and gel images were recorded. The sizes of the PCR products were determined by comparison with the molecular size standard (100 bp ladder; Novagen).

### Microbiology.

Blood cultures were performed in BACTEC TM PLUS aerobic bottles (Becton Dickinson). Identification of the pneumococcal isolates was obtained by conventional techniques (Arbique *et al.*, 2004), including optochin susceptibility and bile solubility; serotyping was only performed by molecular methods.

### Statistical analysis.

All continuous variables were expressed as mean±sd. Fisher’s exact test, Pearson’s chi-square test and the McNemar test were used when appropriate. All *P* values were two-sided, and values less than 0.05 were considered statistically significant. spssx version 10.1 was used to analyse data.

## RESULTS AND DISCUSSION

### Children with and without IPD

Ninety-two children [53 males (57.6 %), 39 females (42.4 %), ratio 1.36] were included in the study, 80 with pneumonia, 8 with meningitis/sepsis and 4 with arthritis. Twenty-two IPD cases [12/53 males (22.6 %), 10/39 (25.6 %) females (*P*=ns)] were diagnosed in biological samples during the 1-year period by RT-PCR and confirmed by *cpsA* positivity during the serotyping procedure with multiplex sequential PCR. Blood samples were positive by molecular methods in all 22 patients; 3/3 CSF samples obtained from patients with meningitis and 2/2 pleural fluid samples obtained from patients with pneumonia were also positive. The distribution of cases with a different disease is presented in Table 2. White blood cell count, neutrophil percentage and C-reactive protein did not differ between IPD and non-IPD patients (respectively 19 956±10 168 vs 21 447±10 378 *P*=ns; 73.1±25.6 vs 76.3±18.4 *P*=ns; 16.3±12.5 vs 12.6±10.4 *P*=ns; normal value for C-reactive protein is <0.3 mg dl^−1^). None of the children with IPD had an underlying disease. All were previously healthy. The clinical outcome was evaluated in all 22 cases: no case of death was recorded but one patient suffered a permanent sequela (partial cranial nerves palsy) after pneumococcal meningitis.

### Serotyping

Molecular methods allowed serotyping (Table 3) in 19/22 patients (86.4 %). The impossibility of serotyping can derive from the presence of serotypes which are not included in the mix used or from a bacterial load below the limit of sensitivity of multiplex PCR. In accord with previously reported data (Corless *et al.*, 2001), in the three patients that could not be serotyped by the multiplex PCR, the cycle number to reach the baseline threshold value in RT-PCR was always higher than 35, suggesting a very low bacterial load. In patients in whom two biological samples [blood+CFS (*n*=3) or blood+pleural fluid (*n*=2)] were available, the same serotype was found in the two different biological samples. Four of the 19 patients had been vaccinated; they had pneumonia due to non-vaccine serotypes. Fifteen children were not vaccinated: 12 (80 %) were infected by vaccine serotypes or by cross-reactive serotypes and 3 (20 %) were infected by non-vaccine serotypes. All children with major diseases (meningitis/sepsis) were not vaccinated and were infected by vaccine serotypes or cross-reactive serotypes. At best, cultural methods would have serotyped 4/22 (18.2 %) patients, thus giving less informative epidemiological data. The use of molecular methods for diagnosis of IPD is well documented (Corless *et al.*, 2001; McAvin *et al.*, 2001; Verhelst *et al.*, 2003; Morrison *et al.*, 2000) but, up to now, pneumococcus typing by molecular methods has been performed only on cultural isolates and not on clinical samples (Pai *et al.*, 2006; Kong *et al.*, 2006). Therefore, the possibility of serotyping was restricted to samples positive in culture. Among the 22 patients with pneumococcal infection diagnosed by molecular methods, 11/22 (2 meningitis, 9 pneumonia) had received antibiotic therapy before hospital admission. The two patients with meningitis had both received a 2 day antibiotic course with cefixime. The nine patients with pneumococcal pneumonia had received antibiotic therapy for a mean duration of 4.11 days (range 1–11 days) with amoxicillin+clavulanic acid (three cases), ceftriaxone (three cases), cefixime (one case), cefixime and teicoplanin (one case). None of the patients who had received antibiotic therapy were positive by cultural methods. Antibiotic therapy is often performed in children with febrile illnesses on an outpatient basis, before hospital admission. For this reason, micro-organism viability may be compromised and cultural methods may be unable to detect invasive pneumococcus infection (Corless *et al.*, 2001; Dalton & Allison, 1968) or other bacterial infection (Kaczmarski, 1997). On the contrary, PCR-based assays, not requiring viable bacteria, can be successfully used with patients after the beginning of antibiotic therapy. We are aware that positive results of molecular tests in blood may be the results of DNA-aemia and not bacteraemia; however, the association of compatible clinical syndromes with positive results of molecular tests in samples obtained from normally sterile sites (blood, CSF, pleural fluid) appears to strongly suggest an invasive bacterial infection.

### Age distribution and incidence of IPD

**Fig. 1. f1:**
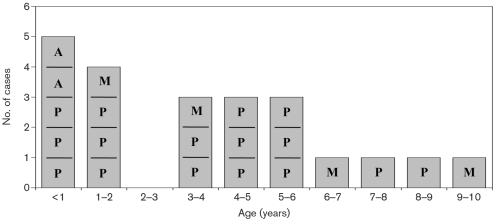
Distribution of 22 cases of IPD according to age and type of disease. A, Arthritis; M, meningitis; P, pneumonia.

**Fig. 2. f2:**
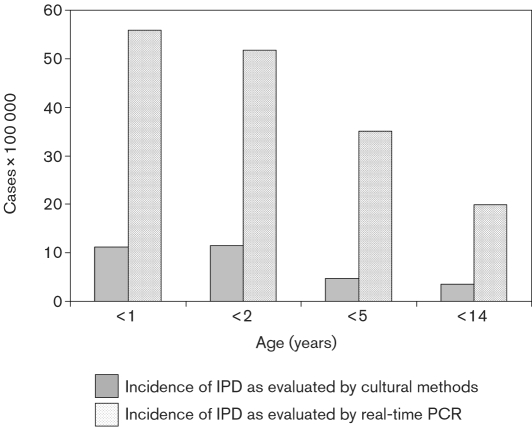
Incidence of IPD as evaluated by cultural or molecular methods.

The mean age of patients included in the study was 63.1±45.2 months (median 52.5 months, range 1–168 months). The mean age of IPD patients was 45.0±33.8 months (median 40.5 months, range 1–120 months) and that of non-IPD patients was 70.3±47.6 months (median 60.0 months, range 1–168 months). IPD patients were significantly younger than non-IPD patients (*P*=0.01, 95 % CI=6.8–44.0). The age distribution of IPD patients is shown in Fig. 1. The Florentine paediatric population below 14 years of age was 110 727, of which 8963 were below 1 year, 17 385 below 2 years of age and 42 756 below 5 years of age (Italian National Institute of Statistics, ISTAT, 2007; http://demo.istat.it/). Larger studies are necessary to give a correct estimate of IPD incidence in relation to paediatric age; however, the present data suggest a higher frequency of IPD when evaluated by molecular methods. The incidence rate for IPD obtained by cultural methods was 3.6/100 000 for children below 14 years, 4.7/100 000 under 5 years, 11.5/100 000 under 2 years and 11.2/100 000 in the first year of life. Using molecular methods, the incidence rate of IPD in children below 14 years of age was 19.9/100 000, the incidence below 5 years of age was 35.1/100 000, below 2 years was 51.8/100 000 and below 1 year was 55.8/100 000 (Fig. 2). The present data confirm that, as in other countries in the world (D'Ancona *et al.*, 2005; Kaltoft *et al.*, 2000; von Kries *et al.*, 2000; Black *et al.*, 2004), the highest incidence of IPD is found in children below 2 years of age and it progressively declines with increasing paediatric age. IPD incidence in children below 2 years obtained in the present study by cultural methods is similar to data previously reported in Italy (D'Ancona *et al.*, 2005) and in other countries in the world (Gendrel *et al.*, 1997; Juvén *et al.*, 2000; Esposito *et al.*, 2004). The present study suggests that IPD incidence in the paediatric population of the Florentine area, evaluated by molecular methods, appears significantly higher, exceeding 50/100 000 in children below 2 years and even higher in children in the first year of life. As demonstrated by Rodríguez-Créixems *et al.* (2003), the incidence of invasive pneumococcal infection is probably even higher than that shown by present results. Indeed patients with fever, often associated with pneumococcal occult bacteraemia (Carvalho *et al.*, 2007), have not been included. Moreover, the present study evaluated only patients admitted to hospital, while patients with pneumonia are usually followed on an outpatient basis. Therefore, the present data are complementary to those obtained by family paediatricians. The main aim of the present study was to evaluate the incidence of invasive pneumococcal infection in the paediatric population, so no data on other aetiological agents responsible for pneumonia cases were collected. The IPD incidence relative to the different diseases studied was 3.6/100 000 for meningitis/sepsis, 1.8/100 000 for arthritis and 14.4/100 000 for pneumonia in children below 14 years of age (Fig. 2). 

### PCR sensitivity

None of the patients negative for pneumococcus by RT-PCR were positive by cultural methods; among the 22 patients who were positive for pneumococcus by molecular methods, 4/22 (18.2 %) were also positive by cultural methods (Table 2). RT-PCR appeared significantly more sensitive than culture in diagnosing IPD (Cohen’s *κ* 0.41; McNemar *P*=0.000008). The four *S. pneumoniae* isolates were penicillin-susceptible. For 18/22 (81.8 %) patients, diagnosis of IPD was only obtained by RT-PCR. In particular, among patients with pneumococcal meningitis or sepsis, 2/4 (50.0 %) were only positive by RT-PCR, while among patients with pneumococcal pneumonia, 15/16 (93.8 %) were only positive by RT-PCR. Of the two patients with arthritis who were positive for *S. pneumoniae* by RT-PCR, one was positive by cultural methods. Increased sensitivity of molecular methods is even more evident in patients with pneumonia than in patients with meningitis/sepsis. For meningitis, the molecular test routinely performed in our laboratory can diagnose both *S. pneumoniae* and *N. meningitidis*, and 8/8 meningitis/sepsis cases could be identified as due to those micro-organisms. The sensitivity of RT-PCR is double that of culture in the diagnosis of meningitis/sepsis and it is about ten times higher in patients with pneumonia. The difference between the two groups of patients can be explained in two ways: (i) patients with meningitis and sepsis usually have a much higher bacterial load, as demonstrated by RT-PCR (data not shown), so that cultural methods, though less sensitive, can detect infection; (ii) patients with meningitis/sepsis usually have a rapid progression of the disease, becoming severe in a few hours, so it is less probable that they have received prolonged antibiotic therapy before hospital admission.

RT-PCR and multiplex sequential PCR are very sensitive methods and their use on clinical samples can help in obtaining a closer estimate of the real incidence of IPD. However, when a positive result is obtained in samples considered negative by the established ‘gold standard’ (culture from blood or CSF), it might be speculated that this is due to poor specificity of PCR rather than improved sensitivity. For this reason, for every sample, we used two different targets from two unrelated genes, *lytA* (known to be extremely sensitive and specific) (Carvalho *et al.*, 2007) in RT-PCR and *cps* in sequential multiplex PCR. Positive results from both targets in the same sample enhance confidence in the sample positivity. Moreover, more than 80 % of the samples could also be serotyped using specific primers for each serotype, different from those for *cps* and *lytA*. At present, cultural methods remain absolutely necessary. First, cultural methods can give important data on antibiotic resistance and individual pathogens which are not included in molecular protocols. Moreover, in some cases, the molecular methods described in the present paper cannot distinguish between serotypes within the same serogroup (i.e. 6A/6B) and more expensive and time-consuming molecular tests would be necessary (Pai *et al.*, 2005). Even if discriminating between serotypes may sometimes be irrelevant for a clinical approach, because a vaccine-induced cross-protection is well known (Vakevainen *et al.*, 2001), distinguishing between serotypes is undoubtedly useful for correct epidemiological evaluation.

At present, case definition of IPD in many countries is based on positive results of standard cultural methods (Pebody *et al.*, 2006). Given that, in many cases, molecular methods show a higher sensitivity (Corless *et al.*, 2001; Van Gastel *et al.*, 2007; Lahti *et al.*, 2006), it could be suggested that case definition should also include diagnosis performed by molecular methods. Indeed, inclusion of molecular assays in the diagnostic tests would improve the diagnostic ability and case ascertainment of invasive bacterial infections.

In conclusion, even though larger studies are required to confirm the present results on IPD incidence, this study demonstrates, for the first time, that an association between RT-PCR and multiplex sequential PCR can be used directly on biological samples for diagnosis and serotyping of IPD. It is likely that similar methods can be used to develop PCR assays able to detect all clinically relevant pneumococcal serotypes that may be found in IPD. The method will be helpful in epidemiological evaluation of circulating serotypes and, consequently, in making correct decisions about vaccination. Molecular methods will also be helpful, even after mass vaccination starts, in monitoring possible epidemiological changes in serotype distribution.

## Figures and Tables

**Table 1. t1:** Primer and probe sequences for RT-PCR

**Gene**	**Forward primer**	**Reverse primer**	**Probe**
*lytA*	5′-ACGCAATCTAGCAGATGAAGC-3′	5′-TGTTTGGTTGGTTATTCGTGC-3′	5′(JOE)-TTTGCCGAAAACGCTTGATACAGGG-6- carboxytetramethyl-rhodamine (TAMRA)-3′
*ctrA*	5′-GCTGCGGTAGGTGGTTCAA-3′	5′-TTGTCGCGGATTTGCAACTA-3′	6-FAM-^680^-CATTGCCACGTGTCAGCTGCACAT-^657^-6-carboxytetramethyl-rhodamine (TAMRA)-3′
*bexA*	5′-GGCGAAATGGTGCTGGTAA-3	5′-GGCCAAGAGATACTCATAGAACGTT-3′	NED-CACCACTCATCAAACGAATGAGCGTGG-6- carboxytetramethyl-rhodamine (TAMRA)-3′

**Table 2. t2:** PCR and culture results for *Streptococcus pneumoniae* in 92 patients No positivity for other micro-organisms was obtained by cultural methods for any of the samples indicated as negative.

	**Patients (*n*=92)**	**PCR results for *S. pneumoniae n* (%)**	**Culture results for *S. pneumoniae n* (%)**
Arthritis	4	Positive 2/4 (50)	Positive 1/4 (25)
		Negative 2/4 (50)	Negative 3/4* (75)
Pneumonia	80	Positive 16/80 (20)	Positive 1/80 (1.25)
		Negative 64/80 (80)	Negative 79/80 (98.75)
Meningitis/sepsis	8	Positive 4/8 (50)	Positive 2/8 (25)
		Negative 4/8 (50)†	Negative 6/8 (75)‡

*One was positive for *Staphylococcus aureus*. †Four were positive for *N. meningitidis*. ‡One was positive for *N. meningitidis*.

**Table 3. t3:** Serotype distribution in 22 IPD patients according to type of disease and vaccination status PF, pleural fluid.

	**Age (months)**	**Vaccinated**	**Culture result**	**RT-PCR results (gene *lytA*)**			**Serotyping**	
				**Blood**	**CSF**	**PF**	PCR with serotype-specific primers*	**PCR for gene *cps***
Arthritis (*n*=2)	6	No		POS			nt	POS
	10	No	POS	POS			22F	POS
Pneumonia (*n*=16)	85	No		POS			4	POS
	24	No		POS		POS	6A/B	POS
	1	No		POS			6A/B	POS
	22	Yes		POS			19A	POS
	22	No		POS			8	POS
	38	No		POS			8	POS
	4	No		POS			nt	POS
	11	No		POS			14	POS
	70	No		POS			18	POS
	108	No	POS	POS		POS	18C†	POS
	51	No		POS			nt	POS
	55	Yes		POS			1	POS
	71	No		POS			19F	POS
	63	Yes		POS			12	POS
	43	Yes		POS			3	POS
	50	No		POS			19F	POS
Meningitis/sepsis (*n*=4)	19	No	POS	POS			4	POS
	38	No		POS	POS		6A/B	POS
	120	No	POS	POS	POS		23F	POS
	80	No		POS	POS		9V/A	POS

*nt, Non-typeable. †Culture confirmed.

## Abbreviations

- CSF, cerebrospinal fluid
- IPD, invasive pneumococcal disease
- RT, real-time
